# EXPLORING SERIOUS ILLNESS CARE EXPERIENCES AMONG BLACK CHICAGOANS’ CAREGIVERS: A QUALITATIVE INQUIRY

**DOI:** 10.1093/geroni/igae098.2203

**Published:** 2024-12-31

**Authors:** Jocelyn Wilder, Kandis Draw, Kimberly Downing

**Affiliations:** NORC at the University of Chicago, Chicago, Illinois, United States; The Hospice and Palliative Care Foundation, Oakbrook Terrace, Illinois, United States; The Hospice and Palliative Care Foundation, Oakbrook Terrace, Illinois, United States

## Abstract

Palliative and hospice services have shown tangible evidence of enhancing the quality of life, decreasing the likelihood of depression, and extending survival for those receiving palliative care. Black individuals receive referrals later and are less likely to utilize palliative and hospice care; national data estimated that only 7.6% of hospice patients were Black. This qualitative study investigates the nuanced perspectives on serious illness care among Black Chicagoans through focus groups and in-depth interviews. Employing an inductive thematic approach, we explored the knowledge, attitudes, and beliefs to understand the intricate cultural, familial, and healthcare dynamics that influence serious illness care experiences among caregivers. Our findings reveal diverse and complex narratives surrounding end-of-life care, reflecting the intersection of race, familial expectations, and healthcare systems. Participants shared moving experiences with family and health care providers, shaping their attitudes towards hospice and palliative care. Discussions highlighted limited knowledge of available services regarding access to and utilization of end-of-life care resources. Thematic analysis identified several key domains, including quality of care, decision-making related to death and dying, and the role of race, highlighting the importance of culturally competent care. These findings underscore the need for healthcare providers to adopt culturally sensitive approaches. This research illuminates the diverse perspectives and experiences within Black communities and informs the development of culturally responsive end-of-life care strategies. Our study contributes to a deeper understanding of the unique challenges and opportunities in providing quality end-of-life care for Black individuals and families.

